# Clinical Features and Outcomes of Surgically Treated Infective Endocarditis in Adults with and Without Congenital Heart Disease: A 12-Year Cohort Study

**DOI:** 10.3390/jcdd13060225

**Published:** 2026-05-25

**Authors:** Shuofang Ren, Lanlin Zhang, Yuanhang Zhai, Sheng Yang, Jianzhou Liu, Xingrong Liu, Shangdong Xu, Guotao Ma, Jun Zheng, Chaoji Zhang

**Affiliations:** Department of Cardiac Surgery, Peking Union Medical College Hospital, Beijing 100730, China; renshuofang@gmail.com (S.R.); lanlinzhang612@163.com (L.Z.);

**Keywords:** infective endocarditis, right-sided endocarditis, cardiac surgery

## Abstract

Background: Adults with congenital heart disease (CHD) are at markedly increased risk of infective endocarditis (IE); however, data comparing clinical characteristics and outcomes in surgically treated IE patients with and without CHD remain limited. This study aimed to evaluate differences in clinical profile, microbiology, complications, and outcomes between these groups. Methods: We conducted a retrospective cohort study of 773 adult patients who underwent surgery for IE at a tertiary center in China between October 2013 and August 2025. Patients were categorized into CHD (*n* = 188) and non-CHD (*n* = 585) groups. Baseline characteristics, microbiological findings, operative data, and postoperative outcomes were compared. Inverse probability of treatment weighting (IPTW) was applied to adjust for baseline differences. Long-term survival was assessed using Kaplan–Meier analysis. Results: Patients with CHD were significantly younger and had fewer cardiovascular comorbidities than non-CHD patients. CHD was associated with a higher prevalence of right-sided and multivalvular infection, whereas non-CHD patients predominantly had left-sided disease. Streptococcus species were the most common pathogens in both groups, with no significant intergroup differences in microbiological profiles. After IPTW adjustment, no significant differences were observed in major postoperative complications, length of stay, or early mortality. Overall and in left-sided IE, long-term survival was comparable between groups, whereas in right-sided IE, patients with CHD appeared to have more favorable long-term survival (HR = 0.17, 95% CI: 0.04–0.66, *p* = 0.01). Conclusions: Despite distinct clinical characteristics, adults with and without CHD undergoing surgery for IE had similar overall outcomes, although CHD was associated with better long-term survival in right-sided IE.

## 1. Introduction

Congenital heart disease (CHD) is present in approximately 1% of newborns. Currently, owing to major improvements in cardiac surgery and perioperative care, up to 97% of all children with CHD are expected to reach 18 years of age or older, resulting in a rapidly expanding population of adults with congenital heart disease (ACHD) [1,2]. These patients are at risk of acquired cardiac conditions, such as heart failure and myocardial infarction, as well as infectious diseases, especially infective endocarditis (IE), which is one of the most severe systemic infectious diseases characterized by high morbidity and mortality [3,4,5,6]. Previous studies have found that patients with CHD carry a more than 50 times higher risk of IE than the general population. This higher risk is probably related to the high prevalence of residual shunts, cyanosis, prosthetic valves, pacemaker leads, and other implanted materials [7,8,9]. However, despite the known relatively high incidence of IE in ACHD, published data comparing IE characteristics and outcomes in patients with and without CHD are sparse [4,10,11]. Therefore, we conducted a 12-year retrospective analysis at a tertiary center in China to compare the clinical profile, microbiological patterns, complications, and outcomes among adult patients undergoing surgery for IE with and without underlying CHD. This study represents one of the most extensive evaluations of surgically managed IE in China and aims to improve clinical care by optimizing local practice.

## 2. Materials and Methods

### 2.1. Study Design

This study was designed as a retrospective cohort study conducted between October 2013 and August 2025.

A total of 773 surgically treated patients were included, comprising 585 (75.7%) without CHD and 188 (24.3%) with CHD. The study protocol was reviewed and approved by the institutional ethics committees of all participating centers, and the requirement for written informed consent was waived owing to the retrospective nature of the study.

### 2.2. Definitions

All patients were diagnosed with infective endocarditis according to the modified Duke criteria. Cases were reviewed by a multidisciplinary team to determine antimicrobial therapy and surgical strategy. Surgical timing was classified as elective, urgent, or emergency; emergency surgery was defined as surgery within 24 h of admission. All operations were performed using standardized cardiopulmonary bypass and myocardial protection protocols by the same surgical team.

Early postoperative mortality was defined as all-cause death within 30 days after surgery. Neurological complications included ischemic stroke, brain abscess, and intracranial infectious aneurysm confirmed by CT or MRI. Embolic events included pulmonary, cerebral, peripheral limb, or other systemic embolization confirmed by imaging or operative findings. Clinical data were obtained from electronic medical records, and follow-up was conducted by structured telephone interviews, with a completion rate of 98.9%.

### 2.3. Data Collection

Comprehensive clinical data were collected through the hospital’s electronic system, including demographic characteristics, pre-existing comorbidities, preoperative laboratory parameters, microbiological findings, echocardiographic characteristics, operative details, and postoperative complications.

### 2.4. Statistical Analysis

Statistical analyses were performed using R software (version 4.5.3). Continuous variables were presented as mean ± standard deviation (SD) or median with interquartile range (IQR), depending on data distribution, and were compared using the Student’s *t*-test or Mann–Whitney U test as appropriate. Categorical variables were expressed as counts and percentages and compared using the chi-square test or Fisher’s exact test.

To adjust for baseline differences between patients with and without congenital heart disease (CHD), inverse probability of treatment weighting (IPTW) based on propensity scores was applied. Propensity scores were estimated using a multivariable logistic regression model incorporating clinically relevant baseline covariates, including demographic characteristics, comorbidities, echocardiographic findings, embolic events, microbiological variables, and surgical timing. Stabilized IPTW weights were calculated, and mild truncation at the 1st and 99th percentiles was performed to improve weight stability and reduce the influence of extreme weights. Covariate balance before and after weighting was assessed using standardized mean differences (SMDs), with an SMD < 0.1 considered indicative of acceptable balance (Supplementary Materials, Table S1).

Weighted analyses were performed using robust variance estimators. Postoperative outcomes were analyzed using weighted logistic regression models, and results were reported as odds ratios (ORs) with 95% confidence intervals (CIs). Long-term survival was evaluated using the Kaplan–Meier method and compared using weighted Cox proportional hazards regression models with robust sandwich variance estimators. Hazard ratios (HRs) and corresponding 95% CIs were calculated.

All statistical tests were two-sided, and a *p* value < 0.05 was considered statistically significant.

## 3. Results

### 3.1. Baseline Characteristics

A total of 773 patients with infective endocarditis (IE) were included, of whom 188 (24.3%) had congenital heart disease (CHD) and 585 (75.7%) did not. The CHD cohort included both repaired and unrepaired congenital lesions. The most common congenital abnormalities were bicuspid aortic valve disease (33.0%), ventricular septal defect (31.9%), and patent ductus arteriosus (17.6%) (Table S2).

Patients in the CHD group were significantly younger than those without CHD (39.8 ± 13.4 vs. 48.6 ± 15.0 years, *p* < 0.001) and had a slightly lower body mass index (21.7 ± 3.6 vs. 22.6 ± 3.7 kg/m^2^, *p* = 0.003). There was no significant difference in sex distribution between the two groups (65% vs. 70%, *p* = 0.2).

With respect to comorbidities, hypertension (29% vs. 11%, *p* < 0.001) and coronary artery disease (11% vs. 5%, *p* = 0.03) were more prevalent in the non-CHD group, while no significant differences were observed in diabetes, pneumonia, chronic obstructive pulmonary disease, heart failure, prior infective endocarditis, or pre-dialysis renal insufficiency. Echocardiographic findings showed no differences in reduced left ventricular ejection fraction (≤50%), perivalvular abscess, or large vegetations. However, left-sided infection predominated in the non-CHD group (91% vs. 59%, *p* < 0.001), whereas right-sided (31% vs. 7%, *p* < 0.001) and bilateral infections (11% vs. 2%, *p* < 0.001) were more frequent in the CHD group.

Regarding valve involvement, aortic valve infection was more common in the CHD group (57% vs. 45%, *p* = 0.002), while mitral valve involvement was higher in the non-CHD group (65% vs. 28%, *p* < 0.001). The CHD group also had higher rates of tricuspid (28% vs. 7%, *p* < 0.001), pulmonary (18% vs. 2%, *p* < 0.001), and multivalvular involvement (26% vs. 18%, *p* = 0.021), whereas prosthetic valve endocarditis was more frequent in the non-CHD group (18% vs. 11%, *p* = 0.029). In terms of embolic events, the overall embolism rate was higher in the non-CHD group (44% vs. 36%, *p* = 0.045), particularly for cerebral embolism (26% vs. 15%, *p* < 0.001), while pulmonary embolism was more common in the CHD group (10% vs. 5%, *p* = 0.008) (Table 1).

### 3.2. Microbiological Findings

Among the 773 patients with infective endocarditis, 596 (77%) had positive blood cultures. The rate of positive blood cultures did not differ significantly between the CHD and non-CHD groups (76% vs. 78%, *p* = 0.6). Regarding pathogen distribution, Streptococcus was the most common organism, accounting for 47% (360 cases), with similar proportions in the CHD and non-CHD groups (51% vs. 45%, *p* = 0.2). Staphylococcus species were the second most common pathogens (16%, 124 cases), also without a significant difference between groups (13% vs. 17%, *p* = 0.2). Among these, *Staphylococcus aureus* accounted for 11% and methicillin-resistant *Staphylococcus aureus* (MRSA) for 5%, with no between-group differences. In addition, Enterococcus and HACEK organisms accounted for 4% and 2% of infections, respectively, with no significant differences between groups. Notably, fungal infections were observed only in the non-CHD group (3% vs. 0%, *p* = 0.017) (Table 2).

### 3.3. Management

Among the 773 patients with infective endocarditis, 101 cases (13%) required reoperation. The proportion of reoperation in the non-CHD group was significantly higher than that in the CHD group (16% vs. 4%, *p* < 0.001). In terms of the timing of the surgery, there was no statistically significant difference between the two groups (*p* = 0.5). Overall, the majority of patients underwent elective surgery (85%), with 86% in the non-CHD group and 82% in the CHD group; the proportion of emergency surgery was similar between the groups (10% vs. 7%; *p* = 0.5). There was also no statistically significant difference in the time of cardiopulmonary bypass (CPB time) between the two groups (150.7 ± 77.0 min vs. 140.7 ± 57.8 min, *p* = 0.12) (Table 3).

### 3.4. Complication

As shown in Table 4, after balancing the baseline characteristics through the inverse probability weighting (IPTW) method, the postoperative complications of patients in the non-CHD group and the CHD group were compared. The results showed that there were no statistically significant differences between the two groups in terms of central nervous system complications, prolonged mechanical ventilation, pneumonia, atrial fibrillation, atrioventricular block, re-thoracotomy for hemostasis, septic shock, ECMO support, and CRRT, among other postoperative complications. Among them, the implantation of pacemakers showed a decreasing trend in the CHD group, but did not reach statistical significance (OR = 0.39, 95% CI: 0.15–1.05, *p* = 0.06). Additionally, no significant difference was observed in low cardiac output syndrome between the two groups (OR = 0.55, 95% CI: 0.28–1.08, *p* = 0.10). In terms of postoperative recovery indicators, there were no significant differences in the length of ICU stay and total hospital stay between the two groups. Moreover, there was no statistical difference in early mortality between the non-CHD group and the CHD group (4% vs. 4%, OR = 0.59, 95% CI: 0.22–1.62, *p* = 0.31).

### 3.5. Outcomes

**Figure 1:**
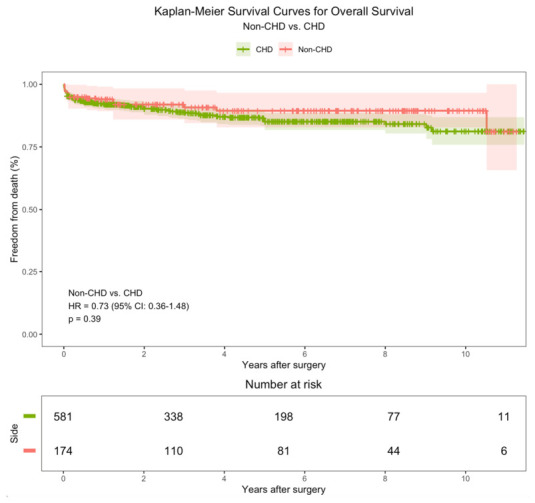
Kaplan–Meier survival curves for overall survival in patients with and without congenital heart disease (CHD).

**Figure 2:**
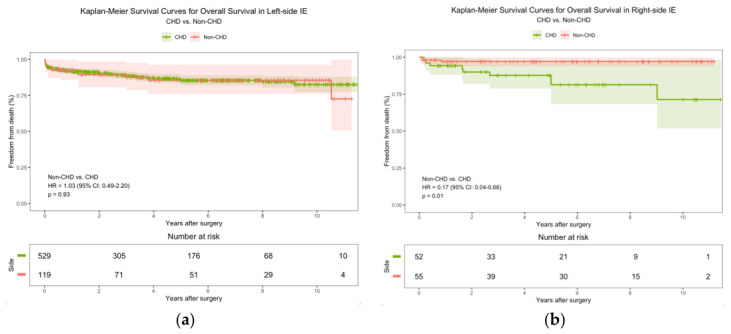
Kaplan–Meier survival curves stratified by infection sidedness in patients with and without congenital heart disease (CHD). (**a**) Kaplan–Meier curves for long-term survival in patients with left-sided infective endocarditis. No significant difference was observed between the CHD group (green) and the non-CHD group (red) (HR, 0.87; 95% CI, 0.39–1.94; *p* = 0.73). (**b**) Kaplan–Meier curves for long-term survival in patients with right-sided infective endocarditis. Patients with CHD (green) had significantly better long-term survival compared with those without CHD (red) (HR, 0.17; 95% CI, 0.04–0.66; *p* = 0.01).

After a median follow-up duration of 5.1 years (IQR, 2.3–7.3 years), with a maximum follow-up of 11.5 years, Kaplan–Meier analysis demonstrated no significant difference in long-term survival between CHD and non-CHD patients in the overall cohort (HR = 0.73, 95% CI: 0.36–1.48, *p* = 0.39) (Figure 1). In the subgroup analysis of left-sided infective endocarditis, there was also no significant difference in the long-term survival rate between the two groups (HR = 1.03, 95% CI: 0.49–2.20, *p* = 0.93). However, in the subgroup of right-sided infective endocarditis, the long-term survival rate of patients in the CHD group was significantly higher than that of the non-CHD group (HR = 0.17, 95% CI: 0.04–0.66, *p* = 0.01) (Figure 2).

To explore the potential influence of temporal changes in surgical practice and perioperative management over the 12-year study period, an exploratory sensitivity analysis stratified by treatment era before and after 2020 was performed. In patients treated before 2020, long-term survival remained comparable between CHD and non-CHD patients (HR = 0.87, 95% CI: 0.39–1.94, *p* = 0.73). Similarly, no significant survival difference was observed among patients treated after 2020 (HR = 0.49, 95% CI: 0.11–2.16, *p* = 0.35) (Figure S1).

## 4. Discussion

Despite growing recognition that patients with CHD constitute a distinct subgroup within the broader IE population, important knowledge gaps remain [6]. Existing comparative studies have largely focused on overall differences in age, comorbidity burden, microbiological profile, surgical rates, and mortality between CHD and non-CHD patients, whereas data specifically addressing patients who undergo surgery for IE remain limited [12,13,14].

### 4.1. Clinical Characteristics

In the present study, surgically treated IE patients with CHD were markedly younger than those without CHD (39.8 ± 13.4 vs. 48.6 ± 15.0 years, *p* < 0.001) and had a substantially lower burden of traditional cardiovascular comorbidities, particularly hypertension (11% vs. 29%, *p* < 0.001) and coronary artery disease (5% vs. 11%, *p* = 0.03). These findings are consistent with prior reports showing that patients with CHD who develop IE are characteristically younger and less comorbid than their non-CHD counterparts, even when the disease progresses to the point of requiring surgical intervention [4,8].

Another notable finding was the striking difference in the anatomical distribution of infection between the 2 groups. In patients without CHD, IE more commonly involved the left heart, whereas patients with CHD were significantly more likely to present with right-sided or bilateral disease, in keeping with prior observations [9]. This divergence likely reflects fundamental differences in the underlying structural and hemodynamic substrate. In CHD, congenital malformations, residual shunts, right ventricular outflow tract abnormalities, prior reparative or palliative procedures, prosthetic material, and high-velocity left-to-right turbulent non-laminar blood flow across abnormal cardiac structures may generate excessive shear stress and endothelial injury, particularly on the right-sided endocardium, thereby facilitating bacterial adherence and subsequent infection [15]. By contrast, in patients without CHD, IE typically develops on left-sided valves in the setting of degenerative valve disease, age-related structural deterioration, and chronically elevated mechanical stress within the high-pressure left-sided circulation [16,17]. Taken together, these findings support the concept that, even within a surgically treated cohort, CHD-related IE arises on a fundamentally different anatomical and pathophysiological substrate than non-CHD IE.

### 4.2. Microbiological Profile

Although the overall microbiological profiles were broadly comparable between CHD and non-CHD patients, several clinically important observations deserve emphasis. Prior studies have suggested that Streptococcus species predominate in CHD-associated IE [12,13,14], whereas *Staphylococcus aureus* is more frequently encountered in patients without CHD [7,9]. However, this pattern has not been entirely consistent across studies. In several Western cohorts, including nationwide data from Denmark, *S. aureus* has emerged as the leading pathogen among patients with CHD, likely reflecting differences in epidemiological context, healthcare exposure, and age-related risk behaviors such as intravenous drug use, body piercing, and tattooing [18,19,20,21]. By contrast, the relatively low prevalence of intravenous drug use in China may partly account for the lower burden of *S. aureus* in our cohort [22]. Within this context, our study showed that the predominant microorganism was Streptococcus species, followed by Staphylococcus species, and there were no significant microbiological differences between CHD and non-CHD patients.

The predominance of streptococcal IE underscores the continued importance of prevention strategies focused on oral health, including regular follow-up, reinforcement of dental hygiene, and appropriate antibiotic prophylaxis in selected high-risk patients undergoing invasive dental procedures. Also, awareness of the local pathogen spectrum is critical for early clinical decision-making, as it may facilitate recognition of clinically meaningful bacteremia and inform empirical antimicrobial selection before definitive culture results become available. Accordingly, even in the absence of statistically significant intergroup differences, the microbiological profile observed in our study provides important insight into both prevention and early management in surgically treated IE.

### 4.3. Outcomes and Prognosis

Another important finding of our present study is that, despite marked differences in baseline characteristics, CHD and non-CHD patients had comparable early and long-term postoperative outcomes. After IPTW adjustment, no significant between-group differences were observed in major postoperative complications, ICU length of stay, total hospitalization duration, and early mortality or overall long-term survival. This finding differs from prior studies showing more favorable outcomes in CHD-associated IE, but likely reflects a fundamental difference in case mix. Most previous reports evaluated unselected IE populations, whereas our cohort was restricted to surgically treated patients. As such, CHD patients in the present study likely represent a selected subgroup with more advanced infection, greater structural complexity, and a higher burden of lesions requiring operative intervention. In this context, the outcomes advantage typically associated with younger age and lower comorbidity burden in CHD patients may be offset by greater disease severity and operative complexity [19]. These findings underscore that the prognostic impact of CHD in IE is highly context-dependent and should be interpreted in light of disease stage and surgical selection, rather than congenital status alone.

Importantly, this apparent equivalence in overall survival masks substantial heterogeneity across subgroups. In stratified analyses, long-term outcomes were similar between CHD and non-CHD patients with left-sided IE, but significantly better in CHD patients with right-sided IE. The absence of a survival difference in left-sided IE suggests that the adverse prognostic impact of left-sided infection may offset the potential protective effect associated with younger age and lower comorbidity burden in CHD patients. This subgroup-specific advantage is biologically plausible. Compared with left-sided disease, right-sided IE is generally associated with more favorable hemodynamics, lower risk of systemic embolization, and less severe ventricular compromise, whereas left-sided IE more often results in heart failure, cerebral embolism and paravalvular extension [23]. In addition, right-sided infection in CHD often involves anatomically well-defined and surgically addressable lesions, such as pulmonary valve or right ventricular outflow tract-related disease, allowing for effective source control and reconstruction. Thus, our findings suggest that the prognostic effect of CHD in surgically treated IE is not uniform but is strongly modified by infection sidedness. Nevertheless, the relatively small number of events in the right-sided IE subgroup resulted in wide confidence intervals, and therefore, this finding should be interpreted cautiously.

From a clinical perspective, these findings underscore that CHD should not be assumed to confer a universal survival advantage once IE progresses to the stage requiring surgery, because surgical selection likely identifies a higher-risk subset of CHD patients than those represented in general IE cohorts. Rather, prognosis appears to reflect the interplay among congenital substrate, disease severity, and infection location. Notably, the favorable outcomes observed in CHD patients with right-sided IE support a proactive surgical strategy in this subgroup when indicated. More broadly, our findings argue for a phenotype-driven approach to risk stratification in IE that incorporates congenital status, infection sidedness, and surgical case selection.

### 4.4. Limitations

Several limitations of this study should be acknowledged. First, this was a retrospective single-center study, which is inherently subject to selection bias and residual confounding despite the use of IPTW adjustment. Second, because the cohort consisted exclusively of surgically treated patients with infective endocarditis, our findings may not be generalizable to the broader non-surgical IE population. Third, the study was conducted in a tertiary referral center in China, and differences in patient characteristics, microbiological epidemiology, and healthcare systems may limit extrapolation to other populations. Fourth, given the long study period spanning 12 years, temporal changes in surgical indications, perioperative management, antimicrobial strategies, and imaging techniques may have influenced clinical outcomes. Nevertheless, exploratory sensitivity analyses stratified by treatment era before and after 2020 demonstrated generally consistent long-term survival patterns between CHD and non-CHD patients across study periods. Finally, although subgroup analyses demonstrated significantly better long-term survival in CHD patients with right-sided IE, the relatively small number of events in this subgroup resulted in wide confidence intervals; therefore, these findings should be interpreted cautiously and require external validation in larger multicenter cohorts.

## 5. Conclusions

Despite distinct clinical characteristics, adults with and without CHD undergoing surgery for IE had similar overall outcomes, although CHD was associated with better long-term survival in right-sided IE.

## Figures and Tables

**Table 1:** Baseline characteristics of the overall population and the comparison of non-CHD IE and CHD IE.

	Overall 773	Non-CHD 585 (75.7%)	CHD 188 (24.3%)	*p*-Value
Baseline Characteristics				
Male	531 (69%)	409 (70%)	122 (65%)	0.2
Age	46.5 ± 15.1	48.6 ± 15.0	39.8 ± 13.4	<0.001
Body mass index, kg/m^2^	22.4 ± 3.7	22.6 ± 3.7	21.7 ± 3.6	0.003
Comorbidities				
Hypertension	193 (25%)	172 (29%)	21 (11%)	<0.001
Coronary artery disease	73 (9%)	63 (11%)	10 (5%)	0.03
Diabetes mellitus	87 (11%)	73 (12%)	14 (7%)	0.06
Pneumonia	68 (9%)	47 (8%)	21 (11%)	0.2
COPD	17 (2%)	15 (3%)	2 (1%)	0.4
Heart failure	236 (31%)	179 (31%)	57 (30%)	>0.9
pre_dialysis	31 (4%)	28 (5%)	3 (2%)	0.052
Previous IE	20 (3%)	19 (3%)	1 (1%)	0.060
Echocardiographic				
LVEF ≤ 50%	32 (4%)	20 (3%)	12 (6%)	0.076
Perivalvular Abscess	107 (14%)	86 (15%)	21 (11%)	0.2
Huge Vegetation	445 (58%)	333 (57%)	112 (60%)	0.5
Affected Valves				
Aortic valve	369 (48%)	261 (45%)	108 (57%)	0.002
Mitral valve	430 (56%)	378 (65%)	52 (28%)	<0.001
Triple valve	93 (12%)	41 (7%)	52 (28%)	<0.001
Pulmonary valve	44 (6%)	10 (2%)	34 (18%)	<0.001
Multiple valves	156 (20%)	107 (18%)	49 (26%)	0.021
Prosthetic valve endocarditis	126 (16%)	105 (18%)	21 (11%)	0.029
Left-side	645 (83%)	534 (91%)	111 (59%)	<0.001
Right-side	97 (13%)	39 (7%)	58 (31%)	<0.001
Double-side	33 (4%)	12 (2%)	21 (11%)	<0.001
Embolic events				
Overall Embolism	324 (42%)	257 (44%)	67 (36%)	0.045
Cerebrovascular	182 (24%)	154 (26%)	28 (15%)	<0.001
Lung	47 (6%)	28 (5%)	19 (10%)	0.008

Values are reported as *n* (%), median (IQR) or mean (±Standard Deviation). Abbreviations: IE = infective endocarditis; COPD = Chronic Obstructive Pulmonary Disease; eGFR = estimated glomerular filtration rate; LVEF = left ventricular ejection fraction.

**Table 2:** Microbiological findings in surgically treated infective endocarditis patients with and without congenital heart disease.

	Overall 773	Non-CHD 585 (75.7%)	CHD 188 (24.3%)	*p*-Value
Pathogen				
Blood culture positive	596 (77%)	454 (78%)	142 (76%)	0.6
Streptococcus	360 (47%)	264 (45%)	96 (51%)	0.2
Staphylococcus	124 (16%)	99 (17%)	25 (13%)	0.2
*S_aureus*	83 (11%)	66 (11%)	17 (9%)	0.4
MRSA	42 (5%)	36 (6%)	6 (3%)	0.12
Enterococcus	34 (4%)	30 (5%)	4 (2%)	0.081
HACEK	19 (2%)	14 (2%)	5 (3%)	0.8
Fungi	16 (2%)	16 (3%)	0 (0%)	0.017

Values are reported as *n* (%), median (IQR) or mean (±Standard Deviation).

**Table 3:** Comparison of surgical-related data for Non-CHD and CHD.

	Overall 773	Non-CHD 585 (75.7%)	CHD 188 (24.3%)	*p*-Value
Re-operation	101 (13%)	93 (16%)	8 (4%)	<0.001
Surgical timing				0.5
Elective	656 (85%)	501 (86%)	155 (82%)	
Urgent	55 (7%)	41 (7%)	14 (7%)	
Emergency	62 (8%)	43 (7%)	19 (10%)	
CPB, min	148.2 ± 72.9	150.7 ± 77.0	140.7 ± 57.8	0.12

Abbreviations: CPB = cardiopulmonary bypass time.

**Table 4:** Comparison of postoperative complications between Non-CHD and CHD after IPTW; Non-CHD was specified as the reference group.

	Overall 773	Non-CHD 585 (75.7%)	CHD 188 (24.3%)	OR (95 CI%)	*p*-Value
CNS	22 (3%)	18 (3%)	4 (2%)	1.03 (0.25–4.25)	0.96
Long ventilation	166 (21%)	133 (23%)	33 (18%)	1.07 (0.64–1.80)	0.80
Pneumonia	81 (10%)	64 (11%)	17 (9%)	1.89 (0.46–1.72)	0.73
Atrial fibrillation	111 (14%)	92 (16%)	19 (10%)	1.19 (0.66–2.15)	0.57
Atrioventricular block	33 (4%)	25 (4%)	8 (4%)	0.77 (0.31–1.90)	0.59
Re-thoracotomy	44 (6%)	38 (6%)	6 (3%)	1.85 (0.66–5.18)	0.29
Low Cardiac Output Syndrome	61 (8%)	45 (8%)	16 (9%)	0.55 (0.28–1.08)	0.10
Septic shock	33 (4%)	28 (5%)	5 (3%)	1.39 (0.49–3.95)	0.59
ECMO	15 (2%)	11 (2%)	4 (2%)	0.60 (0.15–2.44)	0.48
Pacemaker	20 (3%)	12 (2%)	8 (4%)	0.39 (0.15–1.05)	0.06
CRRT	85 (11%)	69 (12%)	16 (9%)	1.13 (0.58–2.19)	0.72
Treatment Time					
ICU time, days	4.0 ± 6.3	4.2 ± 7.0	3.2 ± 3.5	0.57 (0.26–1.27)	0.17
Hospital time, days	13.2 ± 11.3	13.5 ± 12.3	12.3 ± 7.3	0.51 (0.10–2.65)	0.42
Early mortality	33 (4%)	26 (4%)	7 (4%)	0.59 (0.22–1.62)	0.31

Values are reported as odds ratios (ORs) with 95% confidence intervals (CIs). Inverse probability of treatment weighting (IPTW) was applied to balance baseline covariates. Non-CHD was specified as the reference group in all weighted logistic regression models. An OR >1 indicates higher odds of the outcome in Non-CHD compared with CHD.

## Data Availability

The datasets generated and/or analyzed during the current study are available from the corresponding author on reasonable request.

## Supplementary Materials

The following supporting information can be downloaded at: https://www.mdpi.com/article/10.3390/jcdd13060225/s1, Table S1: Baseline characteristics before and after IPTW adjustment; Table S2: Distribution of congenital heart disease subtypes in the CHD cohort; Figure S1: Kaplan–Meier survival curves stratified by study era in patients with and without congenital heart disease (CHD). (A) Overall survival after surgery for infective endocarditis in patients treated after 2020. No significant difference in long-term survival was observed between CHD and non-CHD patients (HR, 0.49; 95% CI, 0.11–2.16; *p* = 0.35). (B) Overall survival after surgery for infective endocarditis in patients treated before 2020. Long-term survival was comparable between CHD and non-CHD patients (HR, 0.87; 95% CI, 0.39–1.94; *p* = 0.73).

## Abbreviations

The following abbreviations are used in this manuscript:

CHD	Congenital Heart Disease
CPB	Cardiopulmonary Bypass
CRRT	Continuous Renal Replacement Therapy
MRSA	Methicillin-Resistant Staphylococcus Aureus
ECMO	Extracorporeal Membrane Oxygenation
ICU	Intensive Care Unit
IE	Infective Endocarditis
IPTW	Inverse Probability of Treatment Weighting
