# Supplementary material for: Clinical Features and Outcomes of Surgically Treated Infective Endocarditis in Adults with and Without Congenital Heart Disease: A 12-Year Cohort Study
Source: J Cardiovasc Dev Dis. 2026 May 25;13(6):225. doi: 10.3390/jcdd13060225 (PMC13302113; doi:10.3390/jcdd13060225)
Supplement: Supplementary file 1 [file jcdd-13-00225-s001.zip › jcdd-4287138-supplementary.pdf]

Table S1. Baseline Characteristics Before and After IPTW Adjustment

Variable	SMD Before	SMD After IPTW
Baseline Characteristics		
Male	0.0502	0.0040
Age	0.6231	0.1121
Body mass index, kg/m²	0.2450	0.0745
Comorbidities		
Hypertension	0.1823	0.0245
Coronary artery disease	0.0545	0.0101
Diabetes mellitus	0.0503	0.0115
Pneumonia	0.0314	0.0271
COPD	0.0150	0.0048
Heart failure	0.0028	0.0207
pre_dialysis	0.0319	0.0071
Previous IE	0.0272	0.0099
Re-operation	0.1164	0.0192
Echocardiographic		
LVEF≤50%	0.0296	0.0057
Perivalvular Abscess	0.0353	0.0044
Huge Vegetation	0.0265	0.0237
Prosthetic valve endocarditis	0.0678	0.0189
Embolic events		
Overall Embolism	0.0829	0.0259
Cerebrovascular	0.1143	0.0273
Lung	0.0532	0.0150
Pathogen		
Staphylococcus	0.0363	0.0051
Blood culture positive	0.0207	0.0192
Surgical timing		
Emergency	0.0099	0.0045

Table S2. Distribution of congenital heart disease subtypes in the CHD cohort

CHD subtype	n (%)
Bicuspid aortic valve disease	62 (33.0)
Ventricular septal defect	60 (31.9)
Patent ducts arteriosus	33 (17.6)
Other congenital heart disease	33 (17.6)

Figure S1. Kaplan–Meier survival curves stratified by study era in patients with and without congenital heart disease (CHD).

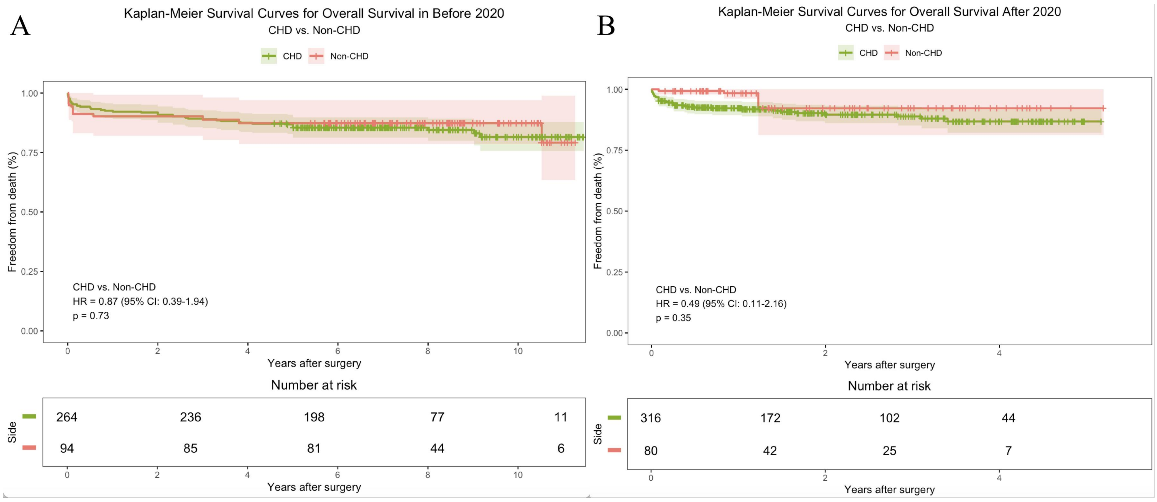

(A) Overall survival after surgery for infective endocarditis in patients treated after 2020. No significant difference in long-term survival was observed between CHD and non-CHD patients (HR, 0.49; 95% CI, 0.11–2.16; P = 0.35).

(B) Overall survival after surgery for infective endocarditis in patients treated before 2020. Long-term survival was comparable between CHD and non-CHD patients (HR, 0.87; 95% CI, 0.39–1.94; P = 0.73).
